# Potential preventive role of Iranian *Achillea wilhelmsii* C. Koch essential oils in acetaminophen-induced hepatotoxicity

**DOI:** 10.1186/1999-3110-55-37

**Published:** 2014-03-24

**Authors:** Abolfazl Dadkhah, Faezeh Fatemi, Shima Ababzadeh, Kambiz Roshanaei, Mahdi Alipour, Bahareh Sadegh Tabrizi

**Affiliations:** 1 Department of Medicine, Faculty of Medicine, Qom Branch, Islamic Azad University, Qom, Iran; 2 Nuclear Fuel Cycle Research School, Nuclear Science and Technology Research Institute, Tehran, IR Iran; 3 Department of Anatomy, Faculty of Medicine, IUMS, Tehran, Iran; 4 Department of Physiology, Faculty of Science, Qom Branch, Islamic Azad University, Qom, Iran; 5 Department of Biochemistry, Faculty of Sciences, Payame-e-Noor University, Tehran, Iran

**Keywords:** *Achillea wilhelmsii* C. Koch essential oils, Metabolizing enzymes, Acetaminophen, Rat, Liver

## Abstract

**Background:**

The essential oil of *Achillea wilhelmsii* C. Koch (100 & 200 mg/kg b.w, i.p) was evaluated against acetaminophen induced hepatic injuries in rats. For this purpose, the activities of cytochrome P450 (CYP450), glutathione s-transferase (GST) and markers of liver injuries (ALT, AST, ALP) together with level of GSH measured analytically in time intervals (2, 4, 8, 16 & 24 h) after treatments confirmed by histophatological consideration in rat livers.

**Results:**

Administration of acetaminophen (500 mg/kg bw, i.p) significantly increased the activity of CYP450 concomitant with increasing the release of ALT and AST. Whereas, GSH level and GST activity were decreased significantly after acetaminophen treatment. Treatment of rats with *Achillea wilhelmsii* essential oils significantly modulate these parameters to normal values. Also, histophatological analysis of liver biopsies was consistent with the biochemical findings.

**Conclusion:**

The data led us to conclude the curative potential of *Achillea wilhelmsii* essential oils against APAP induced hepatic injuries.

**Electronic supplementary material:**

The online version of this article (doi:10.1186/1999-3110-55-37) contains supplementary material, which is available to authorized users.

## Background

Acetaminophen (APAP^a^) is a pain reliever and a fever reducer used extensively to treat many conditions such as headache, muscle aches, arthritis, backache, toothaches, colds, and fevers (Boyer and Rouff 1971; Vermeulen et al. 1992). The main problem with this medication is misuse through intentional or unintentional ingestion of supratherapeutic dosages, which usually lead to hepatic necrosis (Bond et al. 2003).

Acetaminophen is primarily metabolized into sulfate and glucuronide metabolites. A minor pathway through CYP450 had been also reported to yield a highly reactive metabolite, N-acetyl-p-benzoquinonimine (NAPQI). This metabolite is generally stabilized through conjugation with glutathione by glutathione s-transferase (GST) (Henderson et al. 2000) and eliminated via the kidney. Following overdose, and possibly also in extensive and ultrarapid metabolizers, this detoxification pathway becomes saturated, and, as a consequence, NAPQI accumulates (Dong et al. 2000; Hendrickson and Kenneth 2006; Borne 1995). Too much acetaminophen can overwhelm the way the liver normally functions leading to organ failure (Khashab et al. 2007; Hawkins et al. 2007; Larson et al. 2005). Our recent studies together with others indicated that reactive oxygen and nitrogen intermediates, produced by hepatic paranchymal and non-paranchymal cells are believed to be important factors contributing to APAP-induced injury (Dadkhah et al. 2006,2007; Michael et al. 1999; Laskin et al. 1995; Sener et al. 2003). There is increasing interest in the antioxidants of natural origin because they could suppress the oxidative damage of a tissue by stimulating the natural defence system. Consequently, application of natural antioxidants without any side effects for modulation of liver oxidative damages can not be ruled out.

*Achillea wilhelmsii* C. Koch (Asteraceae) wildly grows in different parts of Iran used in traditional medicine for gastrointestinal disorders such as antispasmotic, choleretic, antiulcer, antibacterial and hepatoprotective, antioxidant, anti-inflammatory, anti-allergic, antihypertensive and antihyperlipidemic properties (Nemeth and Bernath 2008; Candan et al. 2003; Yaeesh et al. 2006; Asgary et al. 2000; Fathi et al. 2011; Goldberg et al. 1969; Benedek et al. 2006; Cavalcanti et al. 2006).

Our recent study indicated the *in vitro* antioxidant activity of *Achillea wilhelmsii* essential oils with the p–cymene’ terpinolene and α-thujene as the major compounds (Roshanaei et al. 2012). Continuing this, this research for the first time, pointed out to the effects of *Achillea wilhelmsii* crude extract oils on amending the acetaminophen metabolism aiming the drug toxicity reduction. For this purpose, the most important xenobiotic metabolizing enzymes such as cytochrome P450 (CYP450) and glutathione s-transferase (GST) activities and the level of glutathione (GSH) together with the activities of liver injury markers (Alanine transaminase (ALT), Aspartate transaminase (AST) and Alkaline phosphatase (ALP)) were measured experimentally. Additionally, the histophatological assessment was also accomplished to validate the findings.

## Methods

### Plant preparation

Fresh *Achillea wilhelmsii* C. Koch grown in Iran was collected from Qazvin province. The plant was authenticated by an expert botanist, Prof. Mozaffarian V. Oil extraction was carried out using a Clevenger-type apparatus following component identification by GC/MS analysis. Then, the radical scavenging activity of the oils was measured through radical-scavenging and antioxidant activities (DPPH and ß-Carotene-linoleic acid assays) of the oils (Roshanaei et al. 2012).

### Animal treatments

Male Wistar rats were used throughout this study. Animals were obtained from Pasteur Institute of Iran and maintained in our animal house facilities. Adult animals were 3–4 months of age, weighing 180 ± 20 g. They were maintained on a commercial pellet food and tap water *ad libitum*. Animal studies were approved by the Medical Ethics Committee of Tarbiat Modares University. This Ethics Committee was based on the World Medical Association Declaration of Helsinki (adopted by the 18^th^ World Medical Assembly, Helsinki, Finland, June 1964).

The animals were divided into 16 groups (n = 5). In negative control group (NC), the APAP vehicle i.e. 400 μl DMSO was only injected. In control group (C), the acetaminophen (500 mg/kg b.w) dissolved in 400 μl DMSO was i.p injected. In treatment groups, the essential oils prepared from the plants at two different doses i.e. 100 and 200 mg/kg b.w were diluted in 400 μl DMSO and injected i.p immediately after acetaminophen administration. In positive control group, the BHT (10 mg/kg b.w) dissolved in 400 μl DMSO was injected i.p immediately after acetaminophen administration.

### Preparation of tissue homogenate and plasma

The heparinated blood samples were collected at different time intervals (2, 4, 8, 16 and 24 h after APAP administration) by heart puncture from all the animals and centrifuged at 3000 × g for 10 min to obtain plasma. Liver samples were immediately transferred to ice-cold containers and homogenized (20% w/v) in the appropriate buffer using a homogenizer (E.L.M 2500). The homogenate was used to measure biochemical parameters.

### Biochemical assays

#### GST activity

Liver cytosolic GST activity were measured spectrophotometrically using CDNB as substrate as described in the instruction of the kit buying from (Biovision, USA).

#### Cytochrome P450 activity

CYP activity was performed on liver preparations according to the procedure described in the kit from the (Enzo Life Sciences, Inc., UK).

#### GSH estimation

GSH was estimated in liver homogenate based on the protocol of the purchased kit from (BioVision, Inc., USA).

#### Markers of liver injuries

To confirm the liver function and injury, serum alanine transaminase (ALT), aspartate transaminase (AST) and alkaline phasphatase (ALP) were determined spectrophotometrically according to the procedure described in the kit purchased from the Pars Azmoon, Co, Iran.

### Histophatological studies

The histological changes were quantitatively analyzed by a veterinary pathologist. All animals were sacrificed 24 h after acetaminophen administrations. Small portions of liver were excised from the central lobe and were fixed in 10% buffered formaldehyde solution, embedded in paraffin and sectioned at 6 micrometers. Then, the samples stained with hematoxylin and eosin (H&E) and studied with light microscope for histological analysis.

### Statistical analysis

Data are presented as means ± Standard Error of Mean (SEM). The results were subjected to one-way ANOVA followed by Tukey’s HSD using SPSS (version 19.0) software. Significant levels were defined as P < 0.05.

## Results

### Effects of A. wilhelmsii C. Koch essential oils on hepatic GST and CYP450 activities and GSH level in rats treated by APAP

**Figure 1:**
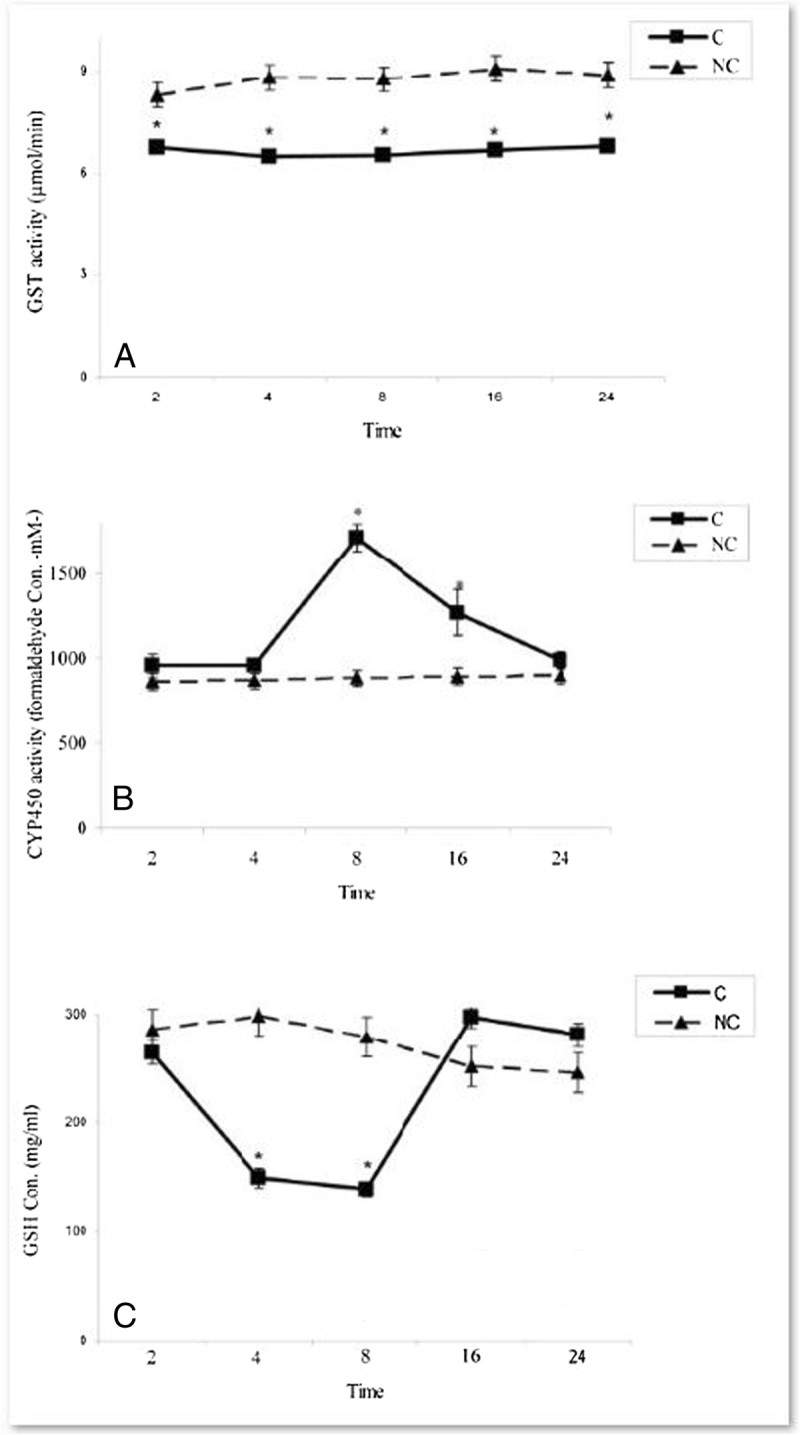
**Time-course changes in xenobiotic metabolizing enzyme activities and GSH level in rats treated with APAP in compare to negative controls. A)** GST activities **B)** CYP450 activities **C)** GSH levels. In negative control group (NC), the APAP vehicle i.e. 400 μl DMSO was only injected. In control group (C), the acetaminophen (500 mg/kg bw) dissolved in 400 μl DMSO was i.p injected. Data are mean ± S.E.M. of five samples obtained from five animals in each group. (*) denote significantly different from the respective negative control group (P < 0.05).

The results in Figure 1A indicated that GST activity was decreased significantly in all time intervals after APAP administration (P < 0.05). Although, the drug administration could significantly affect the GSH level at 4 & 8 h after treatment (P < 0.05) (Figure 1C). CYP450 activity was considerably increased at 8 & 16 h after APAP administration (P < 0.05) (Figure 1B). Thus, it is figured out that APAP administration could change the GST activity at 2-24, GSH level at 4 & 8 h and CYP450 activity at 8 & 16 h after treatments. Depending on these changes, these parameters were considered at 4 & 8 h after APAP treatments in experimental rats treated with essential oils.

**Figure 2:**
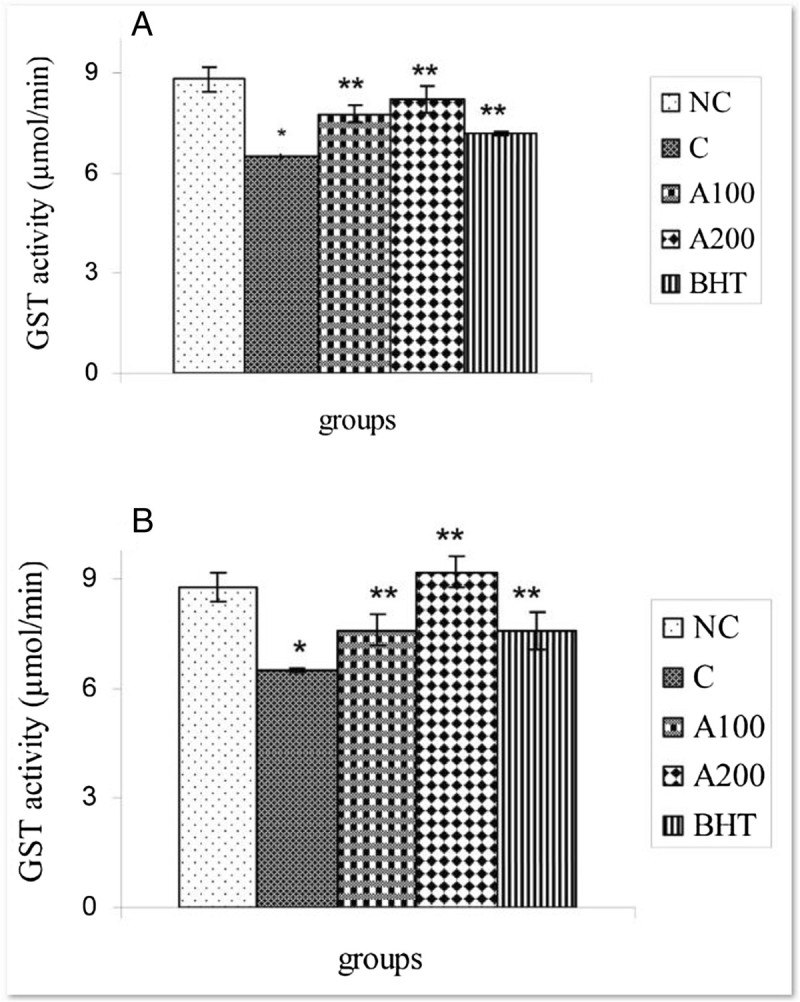
**Effect of** ***A. wilhelmsii*** **essential oils on GST activities after APAP administration. (A)** 4 h after APAP administration **(B)** 8 h after APAP administration. In negative control group (NC), the APAP vehicle i.e. 400 μl DMSO was only injected. In control group **(C)**, the acetaminophen (500 mg/kg bw) dissolved in 400 μl DMSO was i.p injected. In treatment groups (A100 and A200), the essential oils prepared from the plants at two different doses i.e. 100 and 200 mg/kg b.w were diluted in 400 μl DMSO and injected i.p immediately after acetaminophen administration. In positive control group (BHT), the BHT (10 mg/kg b.w) dissolved in 400 μl DMSO was injected i.p immediately after acetaminophen administration. Data are mean ± S.E.M. of five samples obtained from five animals in each group. (*) denote significantly different from the respective negative control group (P < 0.05). (**) denote significantly different from the respective control group (P < 0.05).

**Figure 3:**
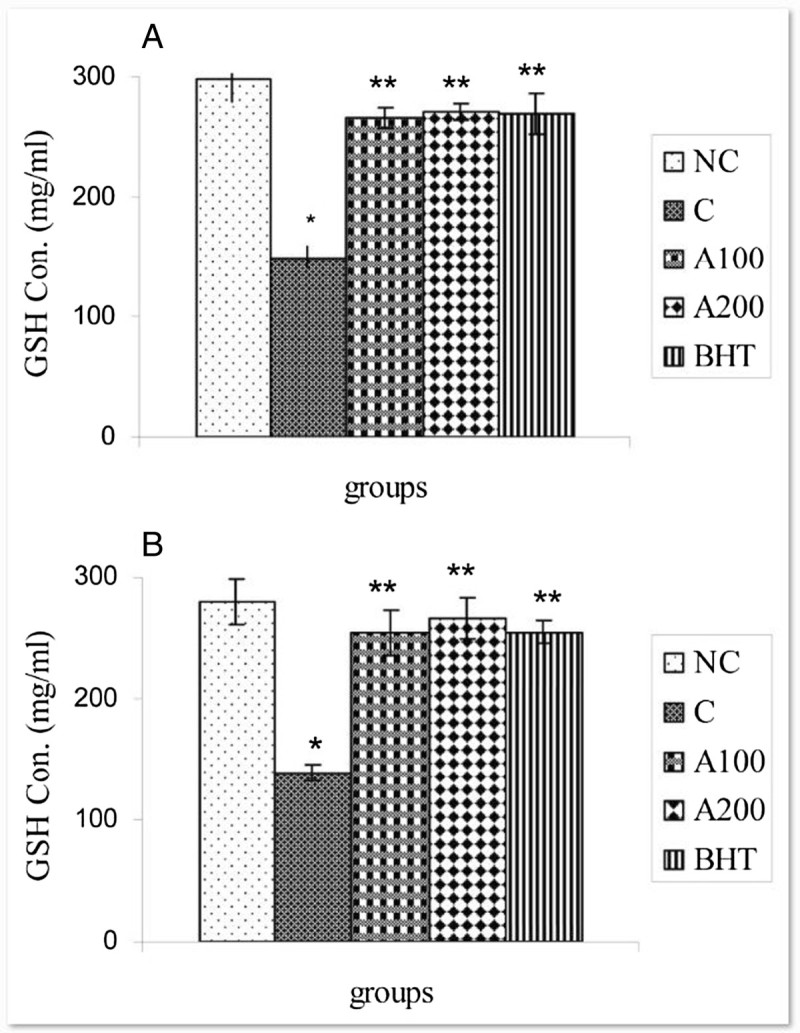
**Effect of** ***A. wilhelmsii*** **essential oils on GSH levels after APAP administration. (A)** 4 h after APAP administration **(B)** 8 h after APAP administration. In negative control group (NC), the APAP vehicle i.e. 400 μl DMSO was only injected. In control group (C), the acetaminophen (500 mg/kg bw) dissolved in 400 μl DMSO was i.p injected. In treatment groups (A100 and A200), the essential oils prepared from the plants at two different doses i.e. 100 and 200 mg/kg b.w were diluted in 400 μl DMSO and injected i.p immediately after acetaminophen administration. In positive control group (BHT), the BHT (10 mg/kg b.w) dissolved in 400 μl DMSO was injected i.p immediately after acetaminophen administration. Data are mean ± S.E.M. of five samples obtained from five animals in each group. (*) denote significantly different from the respective negative control group (P < 0.05). (**) denote significantly different from the respective control group (P < 0.05).

**Figure 4:**
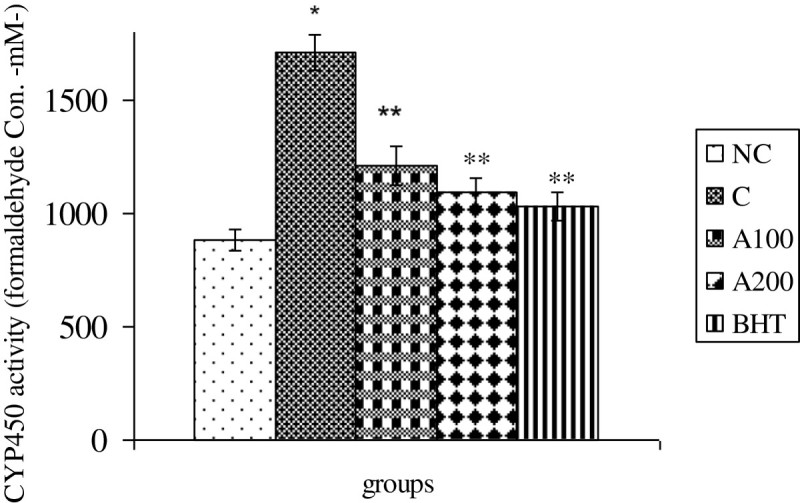
**Effect of** ***A. wilhelmsii*** **essential oils on CYP450 activities 8 h after APAP administration.** In negative control group (NC), the APAP vehicle i.e. 400 μl DMSO was only injected. In control group (C), the acetaminophen (500 mg/kg bw) dissolved in 400 μl DMSO was i.p injected. In treatment groups (A100 and A200), the essential oils prepared from the plants at two different doses i.e. 100 and 200 mg/kg b.w were diluted in 400 μl DMSO and injected i.p immediately after acetaminophen administration. In positive control group (BHT), the BHT (10 mg/kg b.w) dissolved in 400 μl DMSO was injected i.p immediately after acetaminophen administration. Data are mean ± S.E.M. of five samples obtained from five animals in each group. (*) denote significantly different from the respective negative control group (P < 0.05). (**) denote significantly different from the respective control group (P < 0.05).

The data indicated that as reference antioxidant (BHT), the plant essential oils at both doses (100 & 200 mg/kg b.w) could modulate the GST and GSH levels at 4 & 8 h after APAP administrations as compared to those in control groups. (P < 0.05) (Figures 2A-3B). Such this result was also seen in CYP450 activity, decreased significantly 8 h after the essential oils administration at both doses i.e. 100 & 200 mg/kg b.w (Figure 4). There is no significant change in CYP450 activities in all groups 4 h after APAP treatments together with the essential oils (the data not shown).

### Effects of A. wilhelmsii C. Koch essential oils on markers of liver injuries in rats treated by APAP

**Figure 5:**
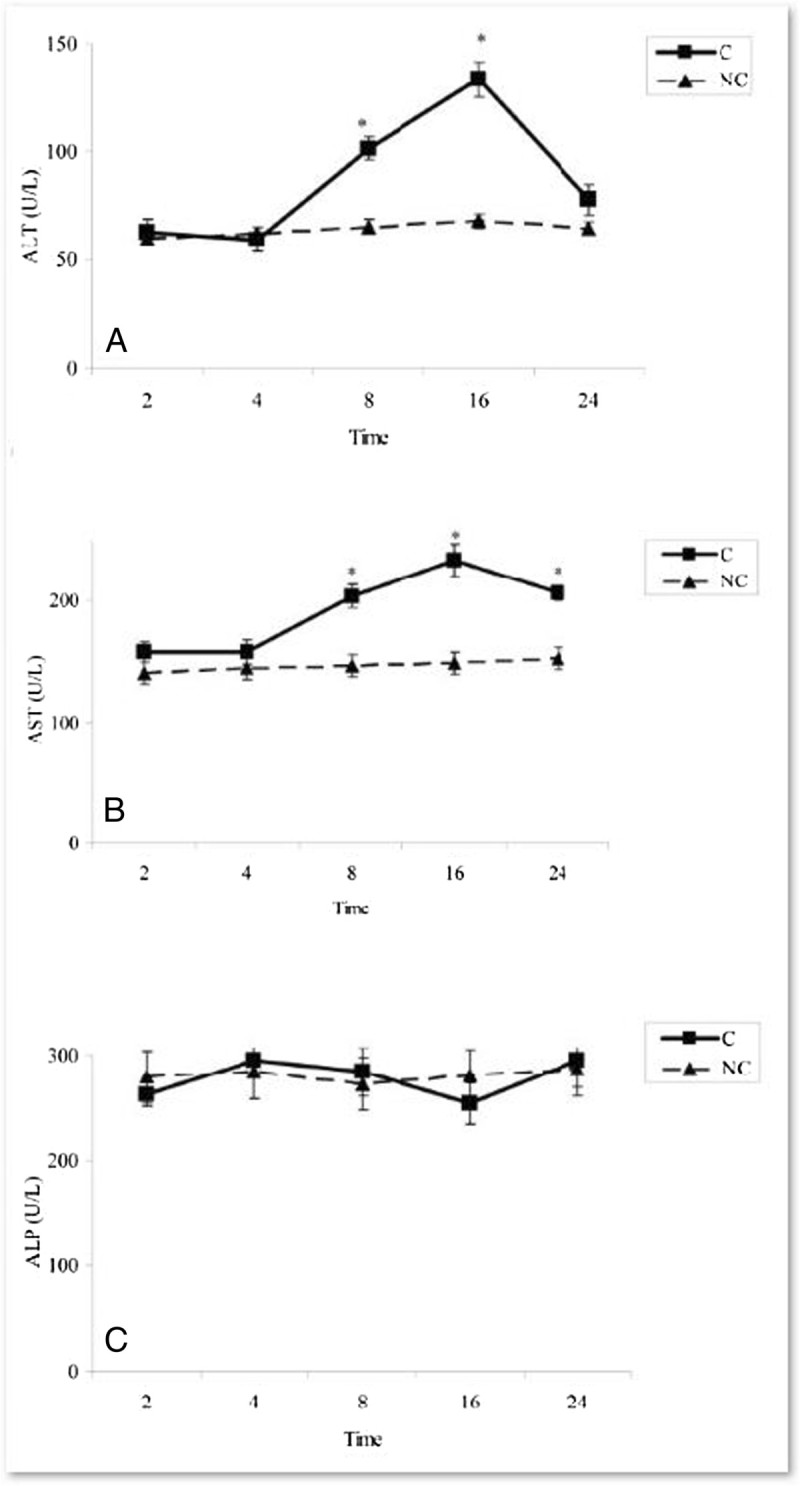
**Time-course changes in hepatic function enzyme activities in rats treated with APAP in compare to negative controls. A)** ALT activities **B)** AST activities **C)** ALP levels. In negative control group (NC), the APAP vehicle i.e. 400 μl DMSO was only injected. In control group (C), the acetaminophen (500 mg/kg bw) dissolved in 400 μl DMSO was i.p injected. Data are mean ± S.E.M. of five samples obtained from five animals in each group. (*) denote significantly different from the respective negative control group (P < 0.05).

**Figure 6:**
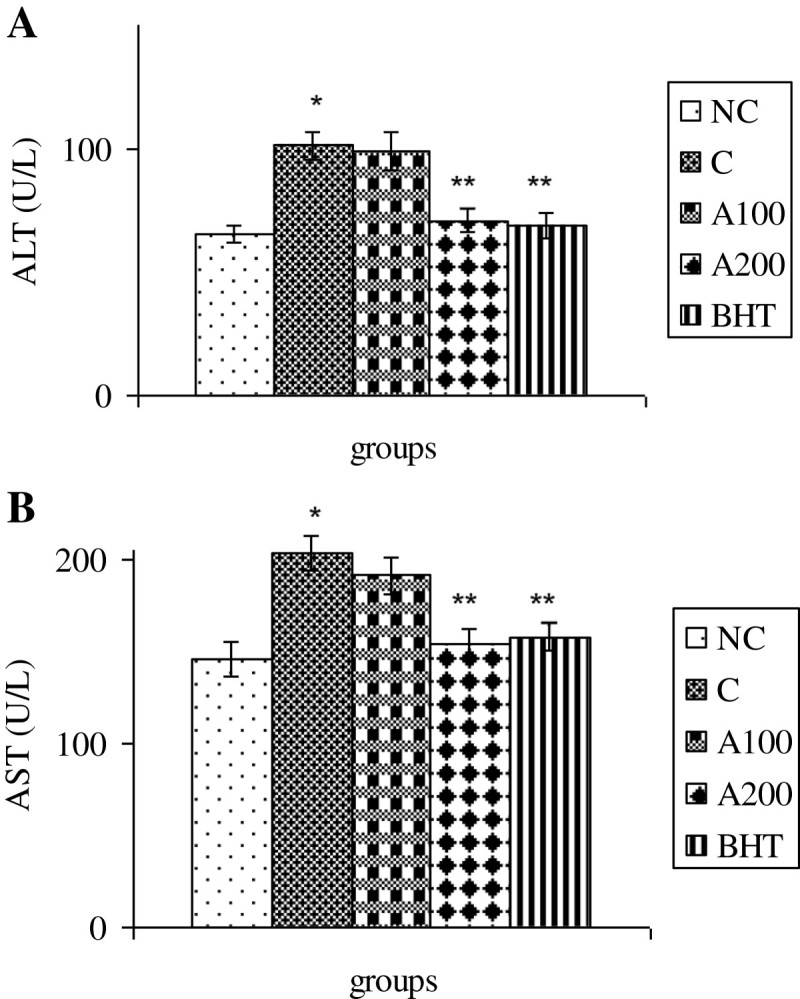
**Effect of** ***A. wilhelmsii*** **essential oils on hepatic function enzyme activities 8 h after APAP administration. (A)** ALT activities **(B)** AST activities. In negative control group (NC), the APAP vehicle i.e. 400 μl DMSO was only injected. In control group (C), the acetaminophen (500 mg/kg bw) dissolved in 400 μl DMSO was i.p injected. In treatment groups (A100 and A200), the essential oils prepared from the plants at two different doses i.e. 100 and 200 mg/kg b.w were diluted in 400 μl DMSO and injected i.p immediately after acetaminophen administration. In positive control group (BHT), the BHT (10 mg/kg b.w) dissolved in 400 μl DMSO was injected i.p immediately after acetaminophen administration. Data are mean ± S.E.M. of five samples obtained from five animals in each group. (*) denote significantly different from the respective negative control group (P < 0.05). (**) denote significantly different from the respective control group (P < 0.05).

The ALT activity was significantly increased at 8 & 16 h after APAP administration (P < 0.05) (Figure 5A). AST activity was also significantly increased at 8 h and remained in high level until 24 h after acetaminophen treatments (P < 0.05) (Figure 5B). As shown in Figure 5C, the ALP activity was not significantly changed in all time intervals as compared to negative control group (P > 0.05). Thus, the ALT and AST activities were examined at 8 h after APAP treatments in experimental rats treated with plant essential oils. The data indicated that the AST and ALT activities were reduced significantly 8 h after treatment of rats with the essential oils (200 mg/kg b.w) and BHT as compare to control groups (P < 0.05) (Figure 6A & B).

### Effects of *A. wilhelmsii* C. Koch essential oils on hepatic histophatological changes in rats treated by APAP

**Figure 7:**
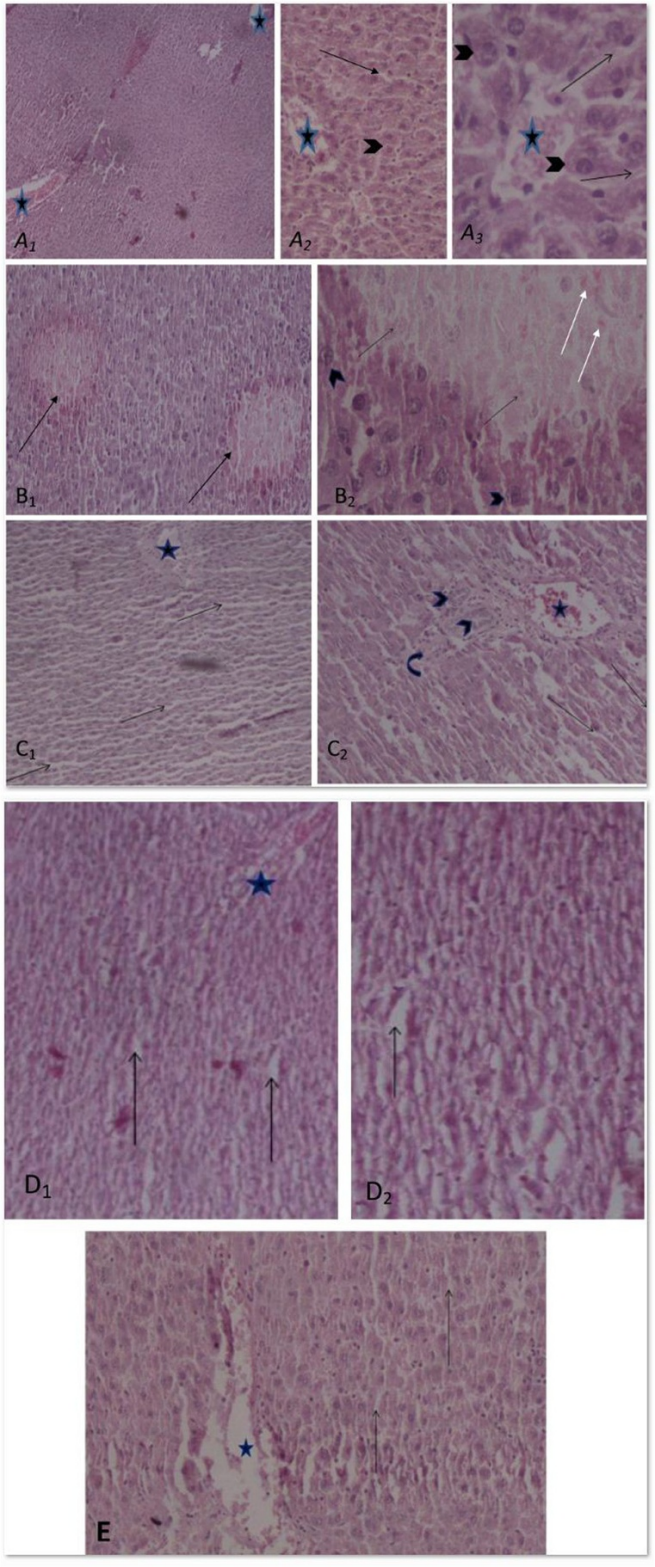
**Effect of** ***A. wilhelmsii*** **essential oils on histopathological changes 24 h after APAP administration.** Light microscopy showed histologic sections of liver of rats from different groups of animals. **A**: Section of liver from negative control group (NC). Normal structure of liver tissue with normal central vein (stars) is detected. Normal sinusoid (arrow) between hepatocyts (arrow head) which form hepatic cords (**A**_**1**_: original magnification ×40, **A**_**2**_: ×100 and **A**_**3**_: ×400). **B**: Section of liver from control group (C) (acetaminophen (500 mg/kg bw)). **B1:** Liver section with necrotic hyalinized aggregation in perilobular zone (arrow) and disarray of hepatic lobule and hepatic cords (**B**_**1**_: original magnification × 100). **B**_**2**_: This section shows hepatocytes necrosis with acidophil bodies (white arrow) and kariolysis (black arrow) in necrosis area. Their nucleuses are comparative with around normal hepatocyte nucleus. The hepatocyte cytoplasm, hepatic sinusoid and hepatic cord are disarray (**B**_**2**_: original magnification ×400). **C**: Section of livers from treatment group (APAP+ 100 mg/kg b.w essential oils) **C**_**1**_: The necrotic regions were not observed and sinusoids (arrows), hepoatocytes and veins (star) are relatively normal (original magnification ×40). **C**_**2**_: Section from preportal and portal space which triad [arteries (bend arrow), bile ducts(arrow head) and portal vein] are normal (original magnification ×100). **D**: Section of livers from treatment group (APAP+ 200 mg/kg b.w essential oil) without of any necrotic zone. Bigger sinusoids (arrow), smaller and more basophilic hepatocytes were observed. The histological structures are relatively disarray (**D**_**1**_: original magnification ×40, **D**_**2**_:×100). **E**: Section of liver from positive control group (BHT (10 mg/kg b.w)) without any necrotic zone, sinusoids (arrows) and hepatocytes around of central vein are normal (star) (original magnification ×100).

Histophatological studies performed on liver biopsies showed normal structure of liver tissue in negative control group (Figure 7A). In liver tissue of rats treated with acetaminophen, minimal 3-8 points of hyalinized cells detected. In some of the cells, wholly white, hyalinized, karyokisis and chromatolysis cytoplasm was also observed. Occasionally, these hyalinized aggregations seems in one parallel row near together affected zonation and distribution of blood in liver (Figure 7B). The necrotic aggregations detected in liver of rats treated with acetaminophen together with 100 mg/kg b.w of *H. Scabrum* essential oils were less than those in control group. The histological structures of sinusoids, hepatocytes, veins, arteries and portal spaces were relatively normal (Figure 7C). No necrotic aggregation was observed in rats treated with acetaminophen plus 200 mg/kg b.w of *H. Scabrum* essential oils. The sinusoids were some more width and hepatocytes were seemed more basophilic. Some little histological distributions were also observed (Figure 7D). Any effect of necrotic aggregation was not illustrated in rats treated with acetaminophen plus BHT. The histological structures of sinusoids, hepatocytes, veins, arteries and portal spaces were perfectly normal (Figure 7E).

## Discussion

The present study indicated that i.p administration of *Achillea wilhelmsii* crude extract oil to APAP-treated rats significantly inhibited hepatic injuries induced by acetaminophen. Our biochemical data indicated that the mechanism by which *Achillea wilhelmsii* essential oils inhibited hepatic toxicity is by modulating the APAP detoxification pathways in the liver which mediate detoxification and metabolic disposal of the intermediate reactive product (NAPQI) leading to inhibition of toxicity process. In this connection, the essential oils (100 & 200 mg/kg b.w) decrease the activities of CYP450 and serum transaminases (ALT and AST) elevated in the liver of APAP treated rats (Figures 4 & 6). Likewise, the decreased hepatic GSH and GST activity in APAP treated rats is compensated by both doses of the essential oils (Figures 2A-3B).

The current findings relating to the effects of *Achillea wilhelmsii* essential oils on APAP-induced hepatic detoxification enzymes are consistent with those of several previous reports that medicinal plants exert their protective effects by modulating the reactive metabolite in the liver (Liu et al. 1995; Kim et al. 2004; Oliveira et al. 2005). Suppression of CYP450 by the essential oils (Figure 4) diminished the formation of APAP reactive metabolite which is leaded to its lower toxic effects (Dong et al. 2000; Hendrickson and Kenneth 2006; Borne 1995).

On the other hand, many studies have indicated that decreased toxicity can be achieved through the modulation of the GST activity (Henderson et al. 2000). GST is a biotransformation enzyme in phase II involved in the detoxification of xenobiotics by conjugating these toxic substances with GSH, ultimately protecting cells and organs against drug-induced toxicity. Reduction of GST activity together with GSH depletion (Figure 1A & C) in liver of APAP-treated rats may be due to its effective role in detoxification of reactive metabolite of APAP (Henderson et al. 2000). Increasing of GST in liver by *Achillea wilhelmsii* essential oils which use GSH as substrate (Figures 2A-3B) might advice to more metabolic disposal of APAP metabolites, resulting in the protection of hepatocytes and simultaneous inhibition of toxicity. On the other hand, it was found that GST and GSH is induced upon oxidative stress (Moghadasian et al. 1996; Toyokuni et al. 1995). In addition, GST, a secondary antioxidant enzyme used in drug detoxification, helps in the radical-scavenging activity of GSH (Kumari and Kakkar 2012). Therefore, the GST induction following essential oil treatments may reflect a decreased oxidative stress arising from the oxygen radical scavenging activity of *Achillea wilhelmsii* essential oils*.*

According to the decline in hepatic GSH content (Figure 1C), it was evident that paracetamol-induced toxicity involved a change in cellular redox status toward a state of oxidative stress. A wide variety of oxidizing molecules such as ROS and/or depleting agents can alter glutathione redox state, which is normally maintained by the activity of GSH-depleting (GPx, GST) and GSH-replenishing (GR) enzymes (Halliwell 1996). Therefore, it can be assumed that decreasing in the GSH concentration might cause the effectiveness of GST and GPx activity to be restricted, as evident by the intensification of lipid peroxidation (Czeczot et al. 2006). The APAP induced depletion of GSH was restored by essential oil treatment (Figure 3A & B), which supported the involvement of exogenous administration of antioxidants in modulation of GSH metabolism. Thus, *Achillea wilhelmsii* essential oils may play a key role against APAP intoxication by influencing the cellular GSH pool.

In the present work, the *Achillea wilhelmsii* essential oil also inhibits APAP induced liver damages as demonstrated by the decreasing in plasma transaminases activities (Figure 6A & B). The extent of hepatic damage is assessed by the level of released cytoplasmic transaminases (ALT and AST) in circulation (Chenoweth and Hake 1962; Sallie et al. 1991). ALP is a transmembrain protein in bile canalicules and releases in blood circulation when the canalicules blocked or disturbed. The maintenance of plasma ALP level in all groups indicates that hepatic damages don’t affect the bile canalicules. The binding of NAPQI with cellular proteins leads to necrosis in liver (Dahlin et al. 1984), which subsequently alters the liver function tests. The observed hepatoprotective effect might be a consequence of the stabilization in the redox state and maintenance of the antioxidant capacity offered by *Achillea wilhelmsii* essential oils. Other studies also confirming these results indicating the protective effect of *Cyperus Scariosus* and *Artemisia absinthium* extract on acetaminophen induced hepatotoxicity have been shown via lowering the respective serum AST and ALT (Gilani and Janbaz 1995a, b). Protective effect of α- and β-amyrin, a triterpene mixture from *Protium heptaphyllum* (Aubl.) March. trunk wood resin, against acetaminophen-induced liver injuries in mice through diminution in oxidative stress and toxic metabolite formation was also reported (Oliveira et al. 2005). One study showed the protective effects of quercetin and curcumin on paracetamol-induced liver injury in rat through mitigation the rise in markers of liver injuries such as AST and ALT (Yousef et al. 2010). In addition, Propolis extract exhibited curative effects by reversing APAP induced alterations in blood biochemical variables, CYP enzymes and markers of oxidative stress (Bhadauria and Nirala 2009). Lupeol when co-administered with APAP effectively reduces oxidative stress and prevents APAP-induced hepatotoxicity by increasing the intracellular defense mechanism consisting of various antioxidant enzymes (SOD, CAT, GST, GR and GPx) (Kumari and Kakkar 2012). These data were confirmed by histophatological examinations (Figure 7) indicating administration of essential oils decreases the oxidative hepatic injuries such as necrotic aggregations in acetaminophen treated rats.

## Conclusion

Our results indicated that *Achillea wilhelmsii* essential oils may have protective role against APAP induced hepatic damages by reducing the formation of the active intermediate APAP metabolite through modulating of acetaminophen-metabolizing enzyme activities, such as GST and CYP450. These data validates the traditional use of plant in hepatic damages.

## Endnote

^a^**a** cetyl-**p** ara-**a** mino**p** henol.

## Authors’ original submitted files for images

Below are the links to the authors’ original submitted files for images. Authors’ original file for figure 1 Authors’ original file for figure 2 Authors’ original file for figure 3 Authors’ original file for figure 4 Authors’ original file for figure 5 Authors’ original file for figure 6 Authors’ original file for figure 7
