# Supplementary figures and images for: Potential preventive role of Iranian Achillea wilhelmsii C. Koch essential oils in acetaminophen-induced hepatotoxicity
Source: Bot Stud. 2014 Mar 24;55:37. doi: 10.1186/1999-3110-55-37 (PMC5432762; doi:10.1186/1999-3110-55-37)

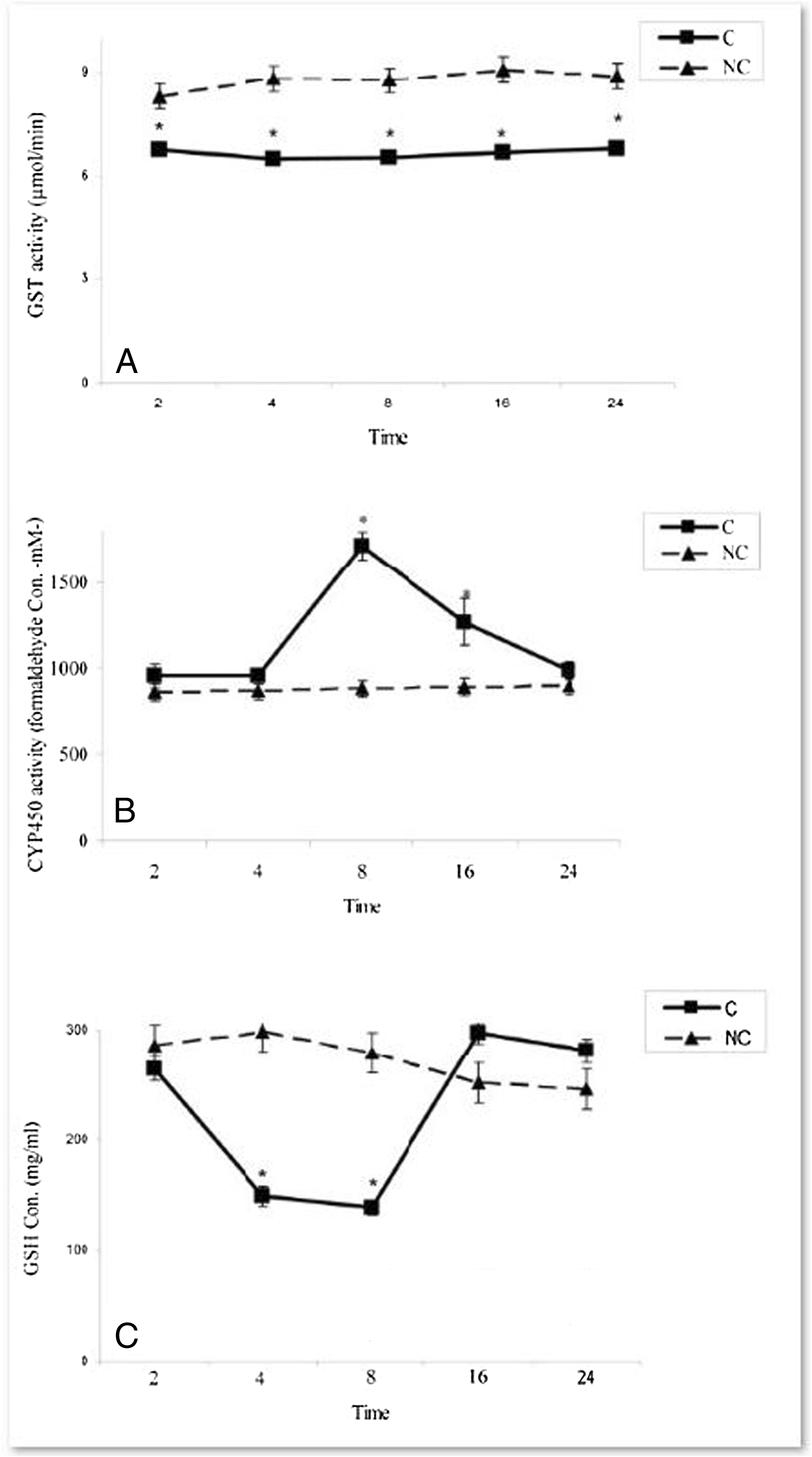

Supplement: Supplementary file 1 — Authors’ original file for figure 1 [file 40529_2012_79_MOESM1_ESM.tiff]

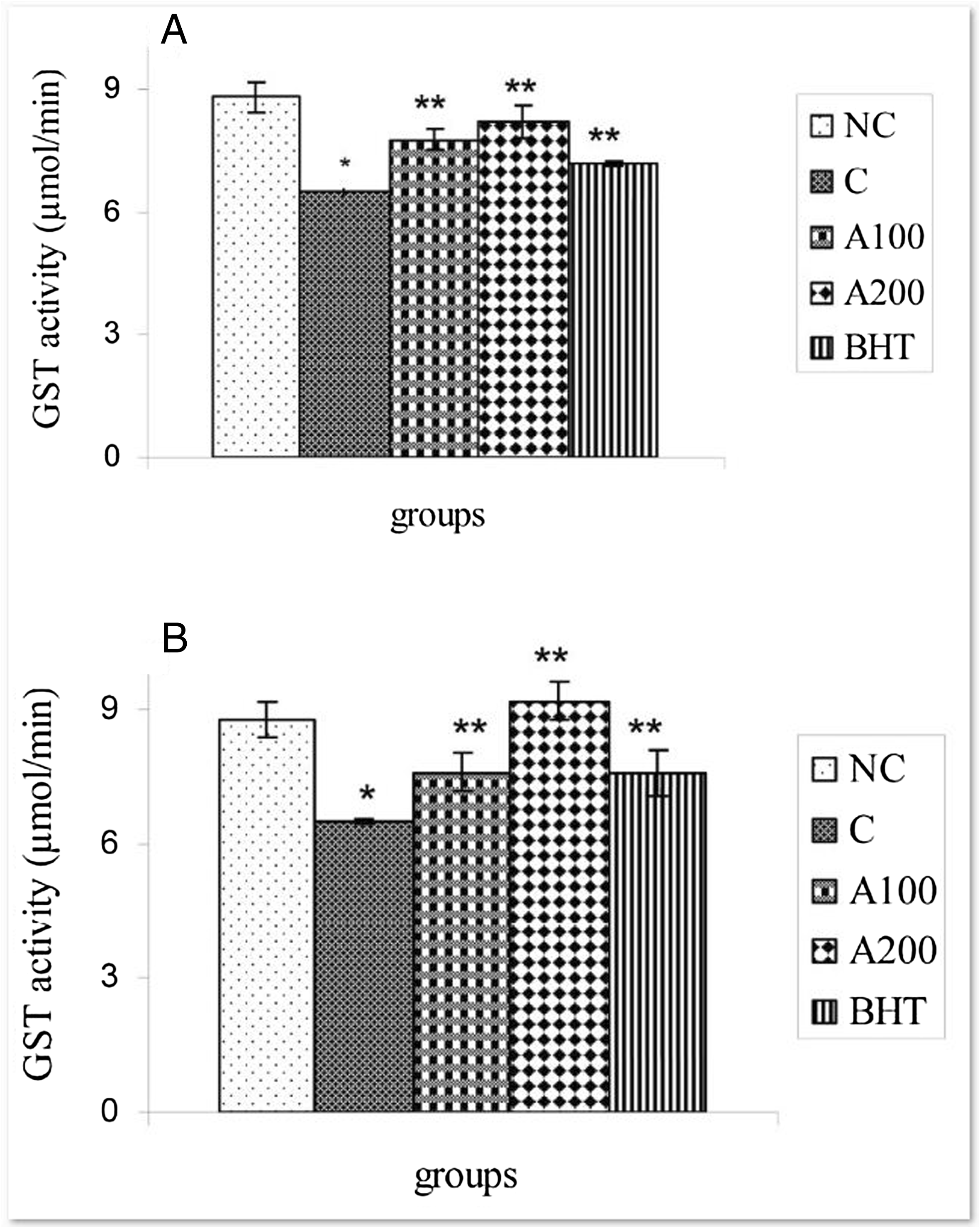

Supplement: Supplementary file 2 — Authors’ original file for figure 2 [file 40529_2012_79_MOESM2_ESM.tiff]

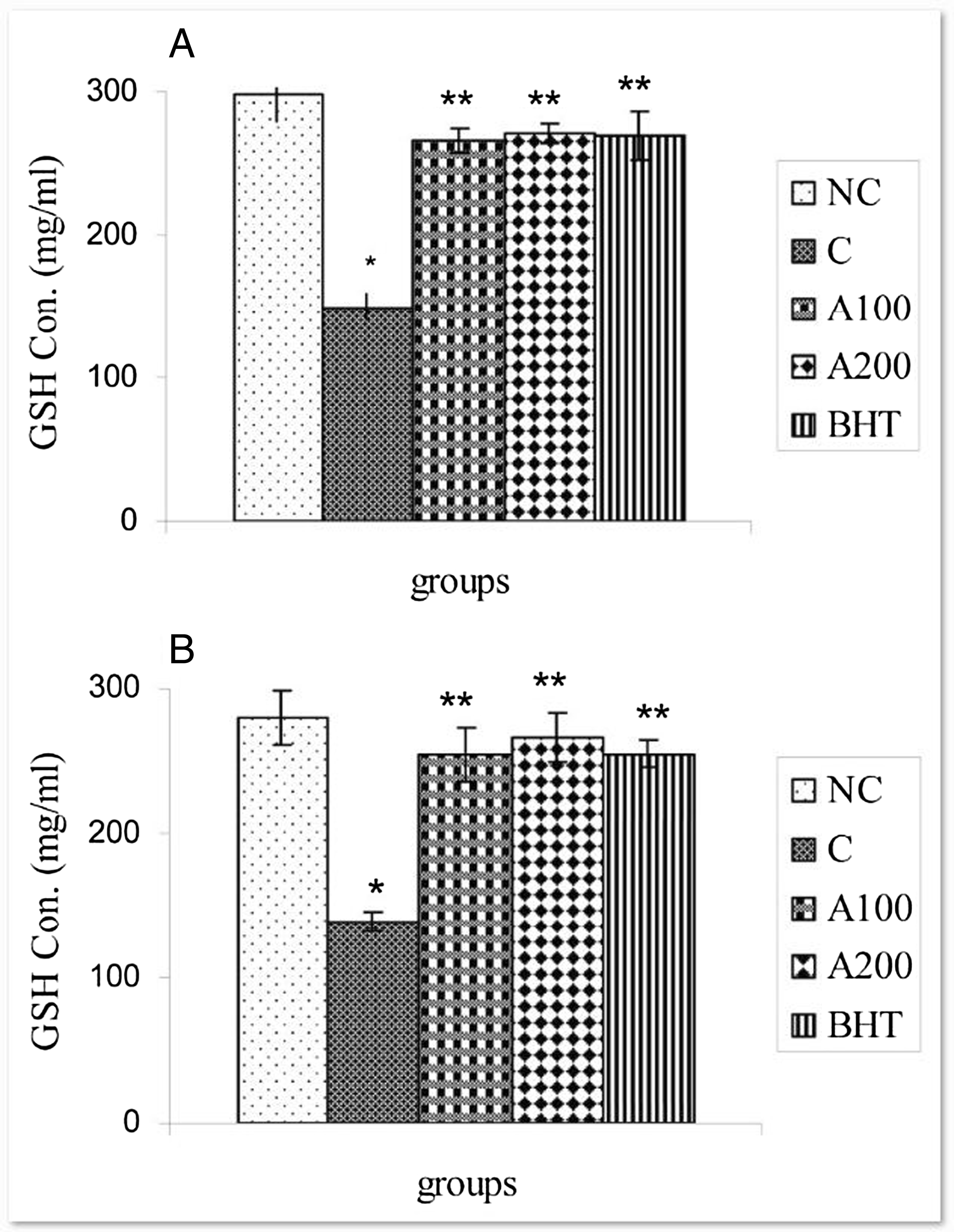

Supplement: Supplementary file 3 — Authors’ original file for figure 3 [file 40529_2012_79_MOESM3_ESM.tiff]

CYP450 activity (formaldehyde Con. -mM-)

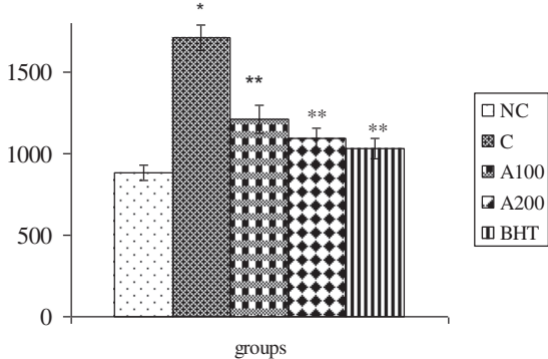

Supplement: Supplementary file 4 — Authors’ original file for figure 4 [file 40529_2012_79_MOESM4_ESM.pdf]

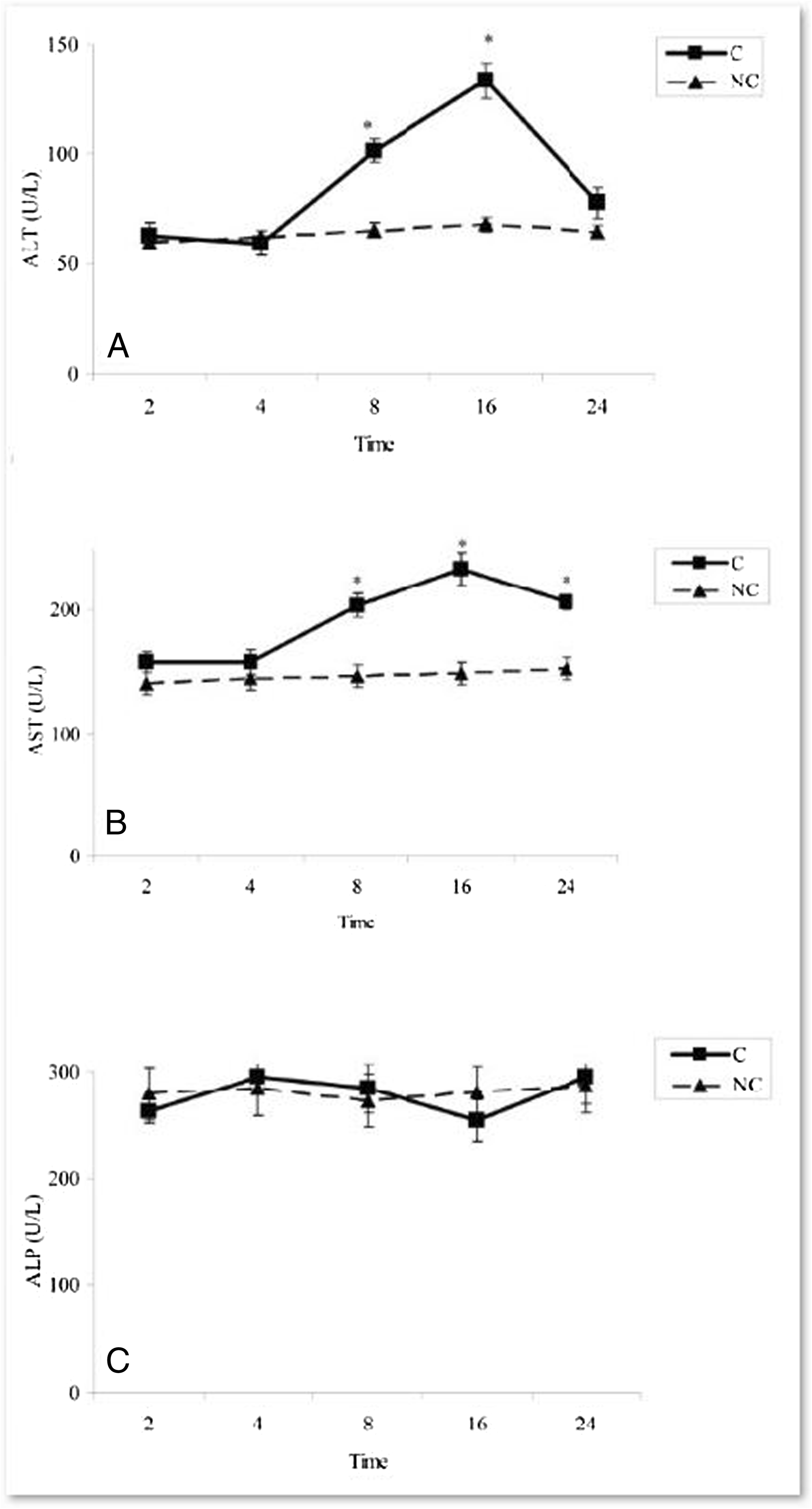

Supplement: Supplementary file 5 — Authors’ original file for figure 5 [file 40529_2012_79_MOESM5_ESM.tiff]

**A**

ALT (U/L)

100

0

groups

□ NC

▣ C

▤ A100

▥ A200

▧ BHT

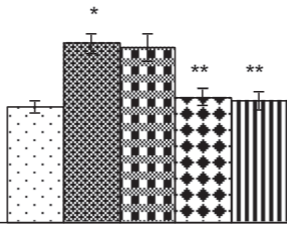**B**

AST (U/L)

200

100

0

groups

□ NC

▣ C

▤ A100

▥ A200

▧ BHT

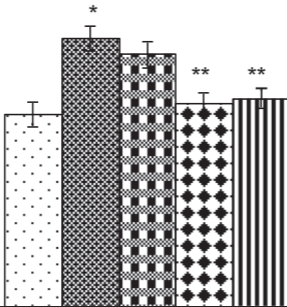

Supplement: Supplementary file 6 — Authors’ original file for figure 6 [file 40529_2012_79_MOESM6_ESM.pdf]

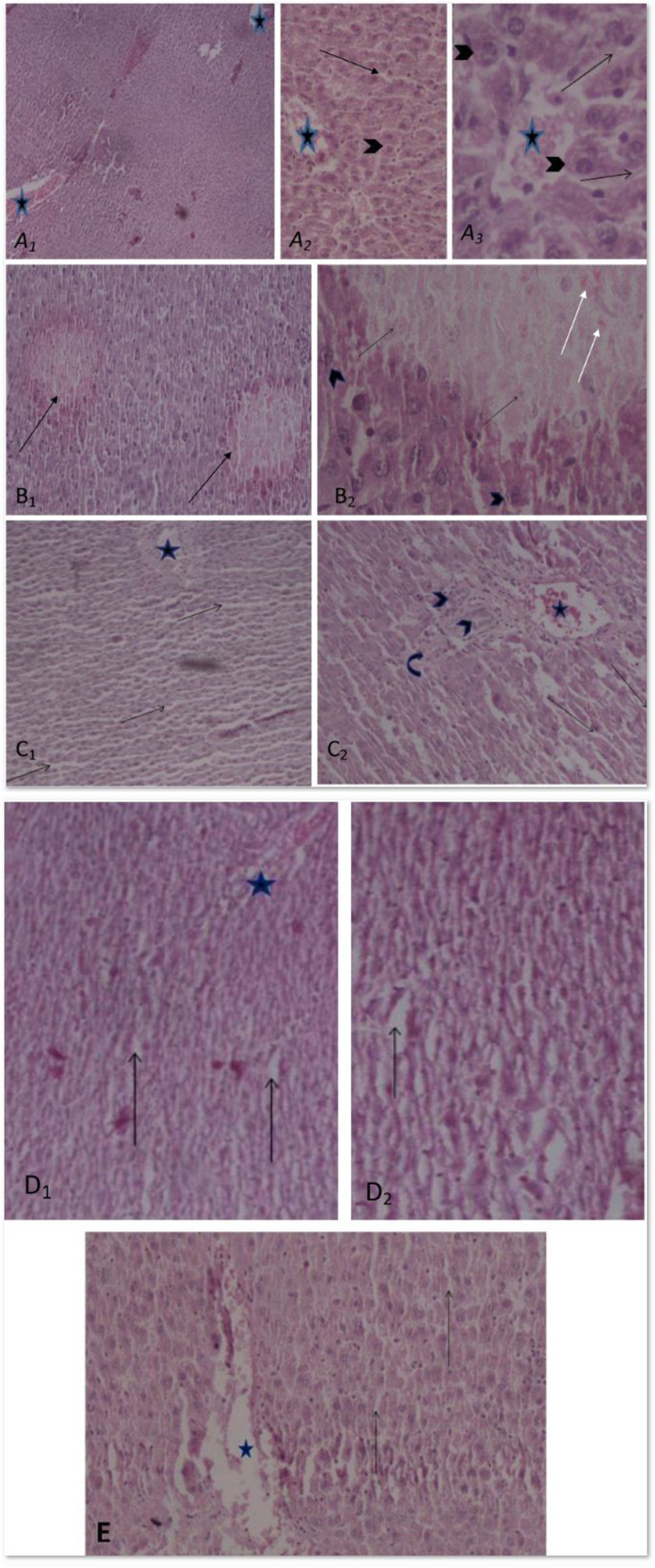

Supplement: Supplementary file 7 — Authors’ original file for figure 7 [file 40529_2012_79_MOESM7_ESM.tiff]
